# Family history scoring system shows Familial Colorectal Cancer Type X syndrome individuals and Amsterdam I individuals have comparable risk for developing colon cancer

**DOI:** 10.1186/1897-4287-9-S1-P35

**Published:** 2011-03-10

**Authors:** Matthew A Strohhacker, James M Church, Janet Shenal, Ellen McGannon

**Affiliations:** 1 Taussig Cancer Institute, Cleveland Clinic, Cleveland, OH, USA; 2 Colorectal Surgery, Sanford R. Weiss Center for Hereditary Colorectal Neoplasia, Digestive Disease Institute, Cleveland Clinic, Cleveland, OH, USA

## Background

A points-based scoring system defines levels of familial risk for colorectal neoplasia. More than 7 points defines a high-risk group that should undergo colonoscopy every 3 years. Family History Scoring was applied to families fulfilling Amsterdam I criteria and familial colorectal cancer type X to compare colon cancer risk.

## Methods

Amsterdam I (AmI) and familial type X (famX) families identified from Jagelman Registry database were scored. Affected probands (AP), unaffected probands (UP), affected sibling (AS), and unaffected sibling (US) of the proband, and a child of each sibling were scored. Median, range, and standard deviation were compared for each syndrome to determine level of risk.

## Results

**Figure 1:**
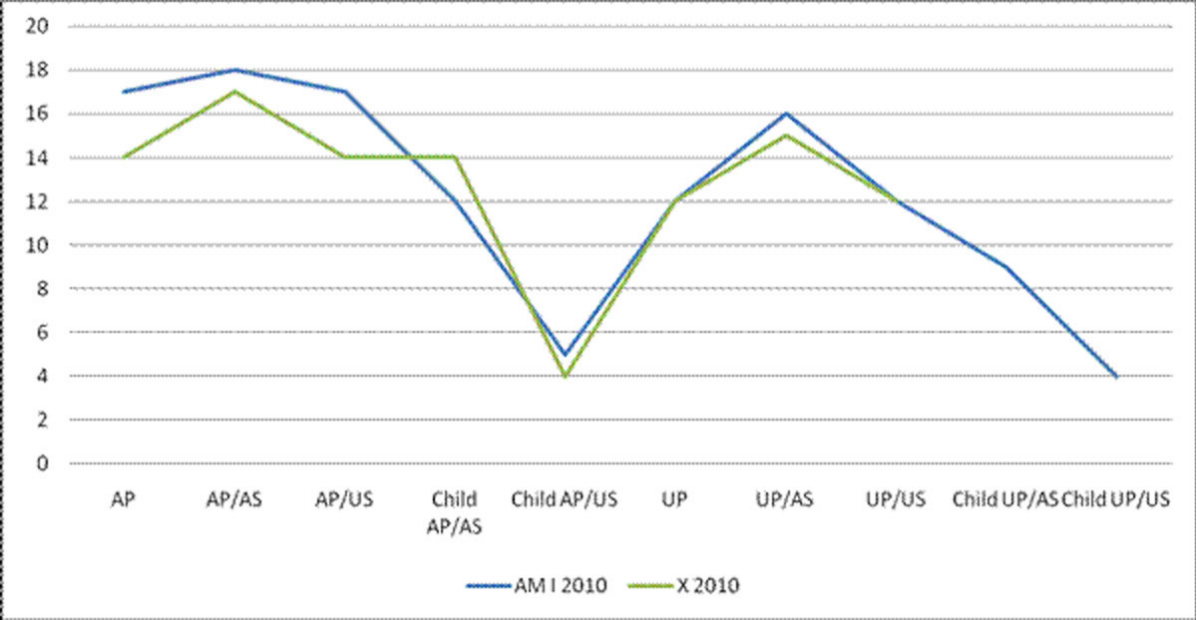
Comparing median score of AmI and famX syndrome using Family History Scoring

200 patients were included. 181 matched AmI criteria with genetic testing, 19 matched criteria for famX. 37 families with an AP and 12 with an UP matched AmI criteria. 4 with an AP and 3 with an UP that matched criteria for famX. Level of risk for the two syndromes were compared (Table 1). Median family history score was 12 points for individuals fulfilling AmI criteria and 14 for famX. Total mean was 12.8 for AmI and 13.5 for famX. Median was compared between the two syndromes (Figure 1), to assess level of risk.

**Table 1:** Comparing level of risk for AmI and famX (X) syndrome using Family History Scoring

		AP	AP/AS	AP/US	AP/AS Child	AP/US Child	UP	UP/AS	UP/US	UP/AS Child	UP/US Child	Total Median
*Median*	AmI	17	18	17	12	5	12	16	12	9	4	**12**
	X	14	17	14	14	4	12	15	12	N/A	N/A	**14**
*Mean*	AmI	18.0	21.0	17.3	13.1	5.2	12.4	15.2	12.5	9.0	4.4	**12.8**
	X	13.5	17.0	13.5	14.0	4.0	11.3	15.0	11.3	N/A	N/A	**13.5**
*Range*	AmI	9-42	14-44	9-30	9-21	4-13	6-19	10-19	6-21	6-12	2-12	
	X	11-15	17	11-15	14	4	7-15	15	7-15	N/A	N/A	
*STDEV*	AmI	6.3	7.0	4.7	3.4	2.0	4.4	3.5	4.7	2.9	3.4	**4.0**
	X	1.9	N/A	1.9	N/A	0.0	4.0	N/A	4.0	N/A	N/A	**1.9**

## Conclusions

Family history scoring system has shown that families fulfilling Amsterdam I criteria and familial type X share similar familial risk level for developing colon cancer. Both show dominant inheritance, but molecular pathways differ.

